# A Manual of Gynæcological Practice

**Published:** 1895-12

**Authors:** 


*A Manual of Gynæcological Practice.* By Dr. A. DÜHRSSEN. Translated and edited from the fourth German Edition by JOHN W. TAYLOR and FREDERICK EDGE, M.D. Pp. xviii., 241. London: H. K. LEWIS. 1895.

This is an excellent little manual for students and practitioners, where in concise and intelligent phraseology we may read with profit the outlines of this branch of professional work. The details of manipulation are clearly expressed; and an account is given of various vaginal operations involving the opening of the peritoneal cavity such as few English text-books contain. This operation, which the author has done so much to bring to perfection, is frequently referred to as vaginal "laparotomy;" but we think a better expression would be vaginal "cœliotomy," a term which is used in other parts of the book.

The mode of expression is occasionally rather loose, and we have noticed some misprints. "Fæcal" is spelt "fœcal" on page 22, "sack" appears for "sac" twice on page 63, and "descencus"

is seen on page 125. The section beginning at page 142 might have been shortened with advantage by omitting those parts of the description of vaginal cœliotomy which have already been given in a previous chapter (pages 54 *et seq.*). The book is well illustrated by numerous plates which elucidate the text, and we can confidently recommend it.

